# *GATA2* mutant variant allele frequency may reflect prognosis in Chinese adult patients with de novo cytogenetically normal acute myeloid leukemia

**DOI:** 10.17305/bb.2024.10244

**Published:** 2024-08-01

**Authors:** Huanying Ren, Minglin Hong, Jingyi Feng, Zhuanghui Hao, Xian Chen, Fengting Liang, Wei Wei, Xuelan Liang, Hongwei Wang, Xiuhua Chen

**Affiliations:** 1 Institute of Hematology, The Second Hospital of Shanxi Medical University, Taiyuan, China

**Keywords:** Acute myeloid leukemia (AML), GATA binding protein 2 (GATA2) gene, variant allele frequency (VAF), prognosis.

## Abstract

Exploration of variant allele frequency (VAF) of *GATA2* mutations (GATA2mut) provides insights into acute myeloid leukemia (AML) prognosis. In this study, we analyzed GATA2mut and co-mutations in 166 Chinese patients with cytogenetically normal AML. This was done through targeted next-generation sequencing (NGS) of 34 genes associated with myeloid leukemia. GATA2mut was identified in 17 (10%) patients as being significantly correlated with co-mutations in *CCAAT*/enhancer-binding protein alpha (*CEBPA*) double mutation (*P* ═ 0.001). We observed that the N-terminal zinc finger domain (*ZF1*) was linked to *CEBPA* mutations, while the C-terminal zinc finger domain (*ZF2*) was associated with Wilms tumor 1 (*WT1*) mutations. It was also noted that patients with GATA2mut had lower platelet counts at diagnosis (*P* ═ 0.032). In the entire cohort, GATA2mut had no significant prognostic impact on overall survival (OS) (*P* ═ 0.762) and relapse-free survival (RFS) (*P* ═ 0.369) compared to patients with GATA2wt. The OS (*P* ═ 0.737) and RFS (*P* ═ 0.894) of the *ZF1* mutation were similar to those of the *ZF2* mutation. Most patients with GATA2mut were classified in the ELN2022 favorable- and intermediate-risk groups. GATA2mut patients in the favorable-risk group were divided into GATA2^High^ and GATA2^Low^ groups using a median cutoff variant allele frequency (VAF) of 40.13%. GATA2^High^ patients were associated with worse OS (*P* ═ 0.031) and RFS (*P* ═ 0.021) than GATA2^Low^ patients. In the intermediate-risk group, the high median VAF of *GATA2* (≥38.51%) had no significant effect on OS and RFS compared with the low median VAF (<38.51%). This study offers new insights into the prognosis of GATA2mut in the favorable-risk group, where VAF can be used as a guide.

## Introduction

The GATA binding protein 2 (*GATA2*) gene, located on human chromosome 3q21, encodes six exons and is a transcription factor of the GATA family, necessary for the regulation of hematopoietic stem cell proliferation and differentiation [1–4]. GATA2mut have been reported in Emberger syndrome [5], chronic myeloid leukemia progressing to acute myeloid leukemia (AML) [6], familial myelodysplastic syndrome (MDS), AML [7–9], and monocytopenia and mycobacterial infection (MonoMAC syndrome) [10]. GATA2mut in AML are nearly always heterozygous and could cooperate with several initiating mutations to promote AML [11]. In addition, the wild-type *GATA2* allele is often epigenetically silenced in AML [11–15].

The *GATA2* gene contains a conserved DNA-binding domain composed of two zinc finger (ZF) domains, which show most somatic GATA2mut in AML [16–21]. Although the prevalence and clinical significance of GATA2mut in adult AML have been extensively studied in recent years, these data are in part conflicting. One study reported in a cytogenetically heterogeneous cohort of 192 adult AML that patients with GATA2mut had significantly better overall and relapse-free survival (RFS) than those without *GATA2* [22]. However, two other studies, also conducted in adult AML patients with cytogenetically heterogeneous backgrounds, found no discernible differences in survival between those with GATA2mut and those with wild-type *GATA2* [23, 24]. These conflicting results may result from the genetic heterogeneity of GATA2-mutated AML, such as different cytogenetic abnormalities and other molecular alterations. Hence, it is crucial to further refine the genetic subclassification to better understand the clinical effect of GATA2mut in adult AML.

In this study, we further refined the biological and prognostic implications of *GATA2* variant allele frequency (VAF) in adult patients with de novo cytogenetically normal AML (CN-AML) through targeted next-generation sequencing (NGS), which may help advance our understanding of the clinical effects of GATA2mut in de novo CN-AML.

## Materials and methods

### Patient information

We retrospectively screened 166 adult patients with newly diagnosed de novo AML and a normal karyotype for GATA2mut at our center between December 2016 and December 2020. These patients had a stored DNA sample from fresh bone marrow (BM) at diagnosis for NGS analyses. Genomic DNA was isolated by the Mini Blood DNA kit (Qiagen, Germany or OMEGA, USA). AML was defined as de novo when no antecedent myeloid malignancy or previous leukemogenic therapy was recorded. Treatment dosage and duration were in accordance with the Chinese Guidelines for the Diagnosis and Treatment of Adult AML (Nonacute Promyelocytic Leukemia). The remission and survival criteria were defined according to ELN2022 recommendations. Relapse was defined only for patients who achieved complete remission (CR) or CR with incomplete hematologic recovery (CRi). The overall survival (OS) was calculated from the time of diagnosis to the time of death or the last follow-up. RFS was calculated from the date of remission until the date of relapse or death from any cause.

**Figure 1.**
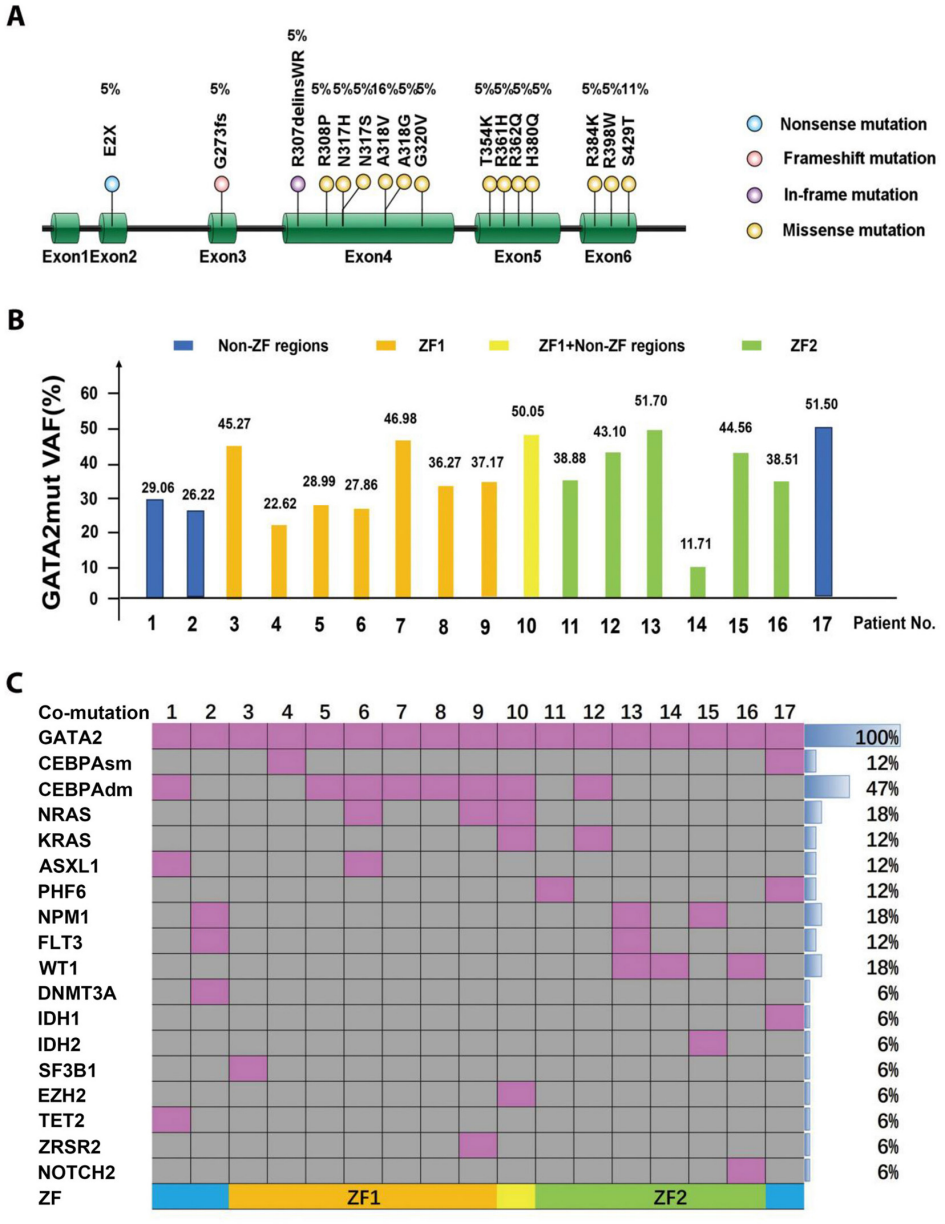
***GATA2* mutations and associated co-mutations.** (A) Scheme of the *GATA2* mutation site; (B) VAF values in 17 patients with *GATA2* mutations. Blue bar indicates that the patient has *GATA2* mutations in non-ZF regions. The orange bar indicates that the patient has GATA2ZF1 mutations. The yellow bar indicates that the patient has GATA2ZF1 and non-ZF region mutations. Green bar indicates that the patient has GATA2ZF2 mutations; (C) Co-mutations and frequency of occurrence in 17 patients with *GATA2* mutations. WT1: Wilms tumor 1; ZF1: N-terminal zinc finger domain; ZF2: C-terminal zinc finger domain; VAF: Variant allele frequency.

### Molecular analysis

Profiling of *GATA2* mutational status and associated co-mutations was achieved by targeted NGS with an AML/MDS/MPN panel of 34 genes known to be frequently mutated in myeloid neoplasms, including *FLT3*, *NPM1*, *KIT*, CCAAT/enhancer-binding protein alpha *(CEBPA)*, *DNMT3A*, *IDH1*, *IDH2*, *TET2*, *EZH2*, *RUNX1*, *ASXL1*, *PHF6*, *TP53*, *SF3B1*, *SRSF2*, *U2AF1*, *ZRSR2*, *NRAS*, *CBL*, *SETBP1*, *ETV6*, *CSF3R*, *NOTHCH2*, *NRAS*, *KRAS*, *SH2B3*, *MLL*, *BOCR*, *BOCR1*, *GATA2*, *MPL*, *WT1*, *PDGFR*, and *JAK2*. The primers for targeted gene amplification are designed in-house, provided by Yuanqi biological medicine technology company (Shanghai, China). The regions analyzed included mutational hotspots or the coding sequence of 34 genes. In brief, 50 ng of genomic DNA was used per reaction. DNA samples from all patients were sequenced and analyzed using a high-throughput sequencing platform, the MiSeq next-generation sequencing instrument (Illumina, San Diego, CA, USA). VAF was calculated using specific DNA sequence variation matching divided by the percentage of the overall coverage of the site. The cutoff VAF was determined using the median VAF of GATA2mut, with a value >5% indicating the presence of a mutation.

### Ethical statement

All patient samples were obtained and analyzed after receiving informed written consent following the Declaration of Helsinki. The study was approved by the Ethics Committee of the Second Hospital of Shanxi Medical University and complied with the Declaration of Helsinki (approval code [2023] YX No. [004]).

### Statistical analysis

Data analysis was performed using SPSS 26.0 software or Graphpad Prism™ 8.01 software. Groups were compared using Pearson’s chi-square analysis or Fisher’s exact test for categorical variables and two independent samples *t*-test or Mann–Whitney *U* test for continuous variables. Survival analysis was estimated using the Kaplan–Meier method, and the log-rank test was used for comparisons. A *P* value of less than 0.05 was deemed statistically significant.

## Results

### Characterization of GATA2mut and their associated co-mutations

GATA2mut were found in 17 of 166 patients (10%). Most patients (*n* ═ 15) showed a single-site mutation in the *GATA2* gene, and two patients (patient no. 4 and no. 10) harbored two different GATA2mut. Mutation types included nonsense mutation, in-frameshift mutation, frameshift mutation, and missense variants, including E2X (5%), R307delinsWR (5%), G273fs (5%), R308P (5%), N317H (5%), N317S (5%), A318V (16%), A318G (5%), G320V (5%), T354K (5%), R361H (5%), R362Q (5%), H380Q (5%), R384K (5%), R398W (5%), and S429T (11%) (Figure 1A). Among them, nine mutations were located in the N-terminal ZF domain (*ZF1*), six in the C-terminal ZF domain (*ZF2*), and four outside of the ZF domains. The *GATA2* VAF values of the 17 patients are shown in (Figure 1B). The median VAF for GATA2mut was 38.51% (11.71%–51.7%). *ZF1* mutations had a VAF value of 22.62%–46.98% with a median of 36.27%, and *ZF2* mutations had a VAF value of 11.71%–51.7% with a median of 40.99%.

All 17 patients with GATA2mut had ≥2 co-mutations, of which seven had two co-mutations, three had three co-mutations, six had four co-mutations, and one had five co-mutations. As shown in Figure 1C, CEBPAdm had the highest mutation frequency (47%), followed by *NRAS* (18%), *NPM1* (18%), *WT1* (18%), *KRAS* (12%), *ASXL1* (12%), *PHF6* (12%), *FLT3* (12%), *DNMT3A* (6%), *IDH1* (6%), *IDH2* (6%), *SF3B1* (6%), *EZH2* (6%), *TET2* (6%), *ZRSR2* (6%), and *NOTCH2* (6%). Other mutated genes were not detected in GATA2mut patients.

### Association of GATA2mut with clinical features and outcomes

Descriptive statistics of clinical parameters demonstrate significant differences between GATA2mut and GATA2wt patients (Table 1). Patients with GATA2mut were diagnosed significantly more often with favorable risk according to the ELN-2022 risk criteria compared with GATA2wt patients (47% vs 29%; *P* ═ 0.123). No differences were detected for the relative proportion of ELN-2022 intermediate risk (41% vs 56%; *P* ═ 0.255) and adverse risk (12% vs 15%; *P* ═ 0.966) between the two groups. Moreover, GATA2mut patients had significantly lower platelet counts (median 31.0 vs 41.5 × 10^9^/L; *P* ═ 0.032) compared with GATA2wt patients. In contrast, there were no significant differences observed in age at diagnosis, sex, white blood cell (WBC) counts, hemoglobin (Hb) level, and BM blast percentages in GATA2mut and GATA2wt patients.

**Figure 2.**
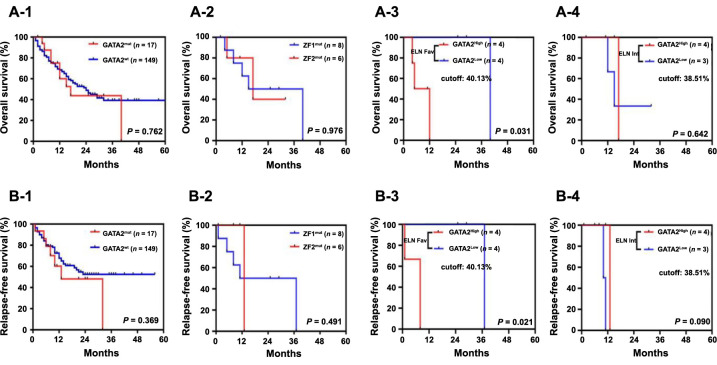
**Prognostic effects of *GATA2* mutations.** Kaplan–Meier survival curves for OS and RFS (A-1 and B-1) in GATA2mut and GATA2wt; (A-2 and B-2) in *ZF1* and *ZF2*; (A-3 and B-3) GATA2^High^ VAF vs GATA2^Low^ VAF in the favorable-risk group; (A-4 and B-4) GATA2^High^ VAF vs GATA^Low^ VAF in the intermediate-risk group. ZF1: N-terminal zinc finger domain; ZF2: C-terminal zinc finger domain; VAF: Variant allele frequency; OS: Overall survival; RFS: Relapse-free survival.

In this cohort, all patients received therapy. A greater number of patients received high-intensity induction in GATA2mut than in GATA2wt groups. In addition, two patients received allogeneic hematopoietic stem cell transplantation (allo-HSCT) in the entire GATA2mut group. Among patients receiving induction therapy, patients with GATA2mut showed a similar CR rate (88% vs 76%; *P* ═ 0.396) to GATA2wt patients. With respect to survival, no significant differences in OS (median: 15 vs 13 months; *P* ═ 0.762) and RFS (median: 8 vs 12 months; *P* ═ 0.369) were detected between GATA2mut and GATA2wt patients. Mutated *GATA2* was significantly associated with CEBPAdm mutations compared to wild-type *GATA2* (47% vs 13%; *P* ═ 0.001) in de novo CN-AML patients. (Table 1; Figure 2).

Most GATA2mut were clustered in the *ZF1* and *ZF2* domains. Considering the potential clinical impact of different domain variants in AML, we further compared the clinical impact between patients with ZF1 and ZF2 mutations. Most clinical features were similar, including age, sex, Hb levels, WBC counts, platelet counts, BM blast percentage, ELN2022-risk, CR rate, OS, and RFS between patients with *ZF1* and *ZF2* mutations. *ZF1* mutations were significantly associated with *CEBPA mutations* (86% vs 17%, *P* ═ 0.029), whereas *ZF2* mutations had a trend associated with Wilms tumor 1 (*WT1*) mutations (50% vs 0%, *P* ═ 0.070; Table 2; Figure 2).

**Table 1:** Clinical characteristics of *GATA2* mutation

**Characteristics**	**GATA2^wt^** **(*N* ═ 149)**	**GATA2^mut^ (*N* ═ 17)**	***P* value**
≥60 years, *n* (%)	51 (34)	4 (24)	0.375
<60 years, *n* (%)	98 (66)	13 (76)	
Male, *n* (%)	81 (54)	9 (53)	0.911
Female, *n* (%)	68 (46)	8 (47)	
WBC (×10^9^/L), median (range)	11.54 (0.8–228.02)	8.74 (0.42–247.86)	0.296
PLT (×10^9^/L), median (range)	41.5 (4–498)	31 (6.4–62)	0.032
HGB (g/L), median (range)	77.5 (45–157.2)	78 (24–118.1)	0.750
BM blast (%), median (range)	0.545 (0.15–0.97)	0.63 (0.2–0.94)	0.768
CEBPAdm, *n* (%)	19 (13)	8 (47)	0.001
High-intensity treatment, *n* (%)	107 (72)	13 (76)	1.000
Low-intensity treatment, *n* (%)	39 (26)	4 (24)	
Allo-HSCT, *n* (%)	19 (13)	2 (12)	1.000
*ELN 2022, *n* (%)*			
FAV-risk	43 (29)	8 (47)	0.123
INTER-risk	83 (56)	7 (41)	0.255
ADV-risk	23 (15)	2 (12)	0.966
*Outcome*			
CR, *n* (%)	113 (76)	15 (88)	0.396
OS (months), median (range)	13 (1–60)	15 (2–40)	0.762
RFS (months), median (range)	12 (1–56)	8 (1–32)	0.369

WBC: White blood cell; PLT: Platelet; HGB: Hemoglobin; BM blast: Bone marrow blast; CEBPAdm: CCAAT/enhancer-binding protein alpha double mutation; Allo-HSCT: Allogeneic hematopoietic stem cell transplantation; ELN: European Leukemia Net; FAV-risk: Favorable risk; INTER-risk: Intermediate risk; ADV-risk: Adverse risk; CR: Complete remission; OS: Overall survival; RFS: Relapse-free survival; GATA2wt: *GATA2* wild-type; GATA2mut: *GATA2* mutations.

**Table 2:** Comparison of clinical features of *ZF1* and *ZF2* mutations

**Characteristics**	**ZF1^mut^** **(*N* ═ 7)**	**ZF2^mut^** **(*N* ═ 6)**	***P* value**
≥60 years, *n* (%)	2 (29)	0	0.462
<60 years, *n* (%)	5 (71)	6 (100)	
Male, *n* (%)	4 (57)	3 (50)	1.000
Female, *n* (%)	3 (43)	3 (50)	
WBC (×10^9^/L), median (range)	13.23 (3.13–97.61)	2.7 (0.42–247.86)	0.073
PLT (×10^9^/L), median (range)	33.4 (6.4–62)	30.5 (24–57)	1.000
HGB (g/L), median (range)	86 (52–118.1)	79.5 (24–114)	0.717
BM blast (%), median (range)	0.74 (0.2–0.94)	0.43 (0.20–0.93)	0.330
*Co-mutation*			
*CEBPA*, *n* (%)	6 (86)	1 (17)	0.029
*WT1*	0	3 (50)	0.070
*ELN 2022, n (%)*			
FAV-risk	5 (71)	2 (33)	0.286
INTER-risk	1 (14)	4 (67)	0.103
ADV-risk	1 (14)	0	1.000
*Outcome*			
CR, *n* (%)	7 (100)	5 (83)	0.462
OS (months), median (range)	25 (8–40)	11 (2–32)	0.737
RFS (months), median (range)	25 (5–37)	8 (1–13)	0.894

WT1: Wilms tumor 1; ZF1: N-terminal zinc finger domain; ZF2: C-terminal zinc finger domain; WBC: White blood cell; PLT: Platelet; HGB: Hemoglobin; BM blast: Bone marrow blast; CEBPA: CCAAT/enhancer-binding protein alpha; ELN: European Leukemia Net; FAV-risk: Favorable risk; INTER-risk: Intermediate risk; ADV-risk: Adverse risk; CR: Complete remission; OS: Overall survival; RFS: Relapse-free survival.

### Clinical impacts of *GATA2* variant allele frequency (VAF)

The impacts of *GATA2* VAF on biological features and patient outcomes were addressed in this cohort. To exclude the clinical effects of co-mutations as the confounding factor, we divided GATA2mut patients into two sets based on ELN2022 risk. To classify double *GATA2* mutant cases into the GATA2^High^ or GATA2^Low^ groups, we used the higher VAF of the double mutations.

In the set of ELN2022 favorable risk (Table 3), we used a median VAF of 40.13% as the cutoff value to further divide GATA2mut patients into two subgroups: GATA2^High^ (*GATA2* VAF ≥40.13%) and GATA2^Low^ (*GATA2* VAF <40.13%). There was only one case with double GATA2mut in each subgroup. GATA2^High^ patients had a higher PLT count (median: 33 vs 15 × 10^9^/L; *P* ═ 0.040) than *GATA2*^Low^ patients. Other biological features, including age, sex, Hb levels, WBC counts, and BM blast percentages, were similar between these two subgroups. In this set, all patients received high-intensity induction. In addition, one patient in the GATA2^High^ subgroup received allo-HSCT. Patients with GATA2^High^ showed a similar CR rate (75% vs 100%; *P* ═ 1.000) to those with GATA2^Low^. In terms of survival, patients with GATA2^High^ had shorter OS (median: 11 vs 25 months; *P* ═ 0.031) and RFS (median: 7 vs 24 months; *P* ═ 0.021) than those with GATA2^Low^ (Table 3; Figure 2).

**Table 3:** Clinical characteristics of GATA2^High^ and GATA2^Low^ in favorable-risk group

**Characteristics**	**GATA2^High^** **(VAF ≥ 40.13%)** **(*N* ═ 4)**	**GATA2^Low^** **(VAF < 40.13%)** **(*N* ═ 4)**	***P* value**
≥60 years, *n* (%)	0	1 (25)	1.000
<60 years, *n* (%)	4 (100)	3 (75)	
Male, *n* (%)	2 (50)	3 (75)	1.000
Female, *n* (%)	2 (50)	1 (25)	
WBC (×10^9^/L), median (range)	69.73 (3.67–247.86)	13.64 (3.13–97.61)	0.486
PLT (×10^9^/L), median (range)	33 (30–36)	15 (6.4–33.4)	0.040
HGB (g/L), median (range)	77 (74–114)	94 (67–118.1)	0.686
BM blast (%), median (range)	0.845 (0.2–0.94)	0.685 (0.2–0.83)	0.343
High-intensity treatment, *n* (%)	4 (100)	4 (100)	–
Low-intensity treatment, *n* (%)	0	0	
Allo-HSCT, *n* (%)	1 (25)	0	1.000
***Outcome***			
CR, *n* (%)	3 (75)	4 (100)	1.000
OS (months), median (range)	11 (4–12)	25 (15–40)	0.031
RFS (months), median (range)	7 (1–8)	24 (10–32)	0.021

VAF: Variant allele frequency; WBC: White blood cell; PLT: Platelet; HGB: Hemoglobin; BM blast: Bone marrow blast; Allo-HSCT: Allogeneic hematopoietic stem cell transplantation; CR: Complete remission; OS: Overall survival; RFS: Relapse-free survival.

In the set of ELN2022 intermediate risk (Table 4), GATA2mut patients were further divided into two subgroups based on a cutoff of a median VAF of 38.51%: GATA2^High^ (*GATA2* VAF ≥38.51%) and GATA2^Low^ (*GATA2* VAF <38.51%). Double *GATA2* mutant cases were not included in this set. All patients received treatment. One patient in the GATA2^High^ subgroup received allo-HSCT. We observed that *GATA2* VAF had no significant impact on biological features or patient survival (Table 4; Figure 2).

**Table 4:** Clinical characteristics of GATA2^High^ and GATA2^Low^ in intermediate-risk group

**Characteristics**	**GATA2^High^** **(VAF ≥ 38.51%)** **(*N* ═ 4)**	**GATA2^Low^** **(VAF < 38.51%)** **(*N* ═ 3)**	***P* value**
≥60 years, *n* (%)	1 (25)	1 (33)	1.000
<60 years, *n* (%)	3 (75)	2 (67)	
Male, *n* (%)	1 (25)	3 (100)	0.143
Female, *n* (%)	3 (75)	0	
WBC (×10^9^/L), median (range)	1.895 (0.42–2.94)	2.59 (0.98–8.74)	0.433
PLT (×10^9^/L), median (range)	28.5 (13–57)	33 (24–41)	0.940
HGB (g/L), median (range)	75.5 (24–104)	76 (52–92)	0.879
BM blast (%), median (range)	0.48 (0.42–0.81)	0.35 (0.24–0.74)	0.557
High-intensity treatment, *n* (%)	3 (75)	1 (33)	0.486
Low-intensity treatment, *n* (%)	1 (25)	2 (67)	
Allo-HSCT, *n* (%)	1 (25)	0	1.000
*Outcome*			
CR, *n* (%)	4 (100)	3 (100)	—
OS (months), median (range)	13 (2–17)	15 (12–32)	0.642
RFS (months), median (range)	8.5 (1–13)	10 (8–11)	0.090

WBC: White blood cell; PLT: Platelet; HGB: Hemoglobin; BM blast: Bone marrow blast; Allo-HSCT: Allogeneic hematopoietic stem cell transplantation; CR: Complete remission; OS: Overall survival; RFS: Relapse-free survival; VAF: Variant allele frequency.

## Discussion

An NGS study of 34 myeloid leukemia-associated genes was performed to assess the mutational profile of *GATA2* in adult de novo CN-AML patients. GATA2mut were found in 10% of adults with CN-AML, and the mutation types were predominantly missense mutations. Somatic GATA2mut mainly cluster in the two ZF domains. In adult AML, *ZF1* mutations predominate, whereas *ZF2* mutations are sporadic. Consistent with this finding, *ZF1* mutations still predominate in adult de novo CN-AML. Previous studies reported that GATA2mut, especially *GATA2 ZF1* mutations [6, 25, 26], often coexist with *CEBPA* mutations, with an incidence of 18%–41% in non-M3 AML patients [1, 27, 28]. Similarly, our cohort showed a significant association between *GATA2 ZF1* mutations and *CEBPA* mutations. Tien et al. reported that ZF1 and ZF2 mutations were mutually exclusive with *KRAS*, *WT1*, *IDH1*, *TP53*, and *ETV6* mutations [25]. However, in our cohort, *ZF2* mutations had a positive correlation with *WT1* mutations.

The clinical impact of GATA2mut on adult de novo CN-AML has not been thoroughly investigated until now. In our cohort with CN-AML, patients with GATA2mut had similar age, sex, Hb levels, WBC counts, and BM blast percentages to those with wild-type GATA2; however, previous reports identified more frequent GATA2mut in AML patients <60 years old than in older patients ≥60 years, including CN-AML and non-CN-AML [22]. However, we observed that CN-AML patients with GATA2mut had significantly lower platelet counts than those with wild-type GATA2. This finding is consistent with some reports. In detail, He et al. reported that patients with CEBPAdm/GATA2mut had lower platelet counts than those with CEBPAsm/GATA2mut [29]. Theis et al.’s cohort study also noted that patients with CEBPAmut/GATA2mut had lower platelet counts than those with CEBPAmut/GATA2wt [30]. Some studies showed better survival with GATA2mut [22, 31], while others found no difference in survival. In this study, we further explored the effect of GATA2mut on the survival prognosis of patients with CN-AML. Our data showed that patients with mutant *GATA2* had similar OS and RFS to patients with wild-type *GATA2*.

Focusing on the clinical and biological implications of different GATA2mut sites, a study in AML patients conducted by Tien et al. reported [25] that most clinical features were similar between ZF1- and ZF2-mutated patients, except that ZF1-mutated patients were younger. With regard to prognostic survival, ZF1-mutated patients had a significantly longer OS than ZF2-mutated patients in the total cohort or patients with normal karyotypes. In contrast, in our cohort, no differences in clinical features, including age, OS, and RFS, were detected between patients with *ZF1* or *ZF2* mutations. These conflicting results might be partially explained by the difference in the association of *ZF2* domain mutations with *WT1* mutations. Further studies are warranted to explore the underlying mechanisms of these differences.

The clinical impact of *GATA2* VAF on patients with AML is unclear. To the best of our knowledge, this is the first study to assess potential associations of *GATA2* VAF with adult de novo CN-AML. To exclude the clinical effects of other molecular alterations, we stratified patients into two groups based on ELN2022 risk. In the favorable-risk set, we subdivided patients at the median *GATA2* VAF (40.13%) into GATA2^High^ and GATA2^Low^ groups; GATA2^High^ had significantly more adverse effects on OS and RFS than GATA2Low. However, in the intermediate-risk set, the high median VAF of *GATA2* (≥38.51%) was similar in OS and RFS to the low median VAF of *GATA2* (<38.51%).

## Conclusion

In summary, we identified that high *GATA2* VAF was associated with an adverse prognostic effect on OS and RFS of adult de novo CN-AML compared with low *GATA2* VAF in the favorable risk subgroup. Our findings highlight potentially novel aspects of the underlying biology of GATA2-mutated CN-AML. However, our study is limited by its retrospective nature and relatively small sample size. Confirmatory research in a larger, prospective cohort would be beneficial in predicting clinical outcomes.

## Data Availability

Data presented in this study are available on request from the corresponding author.
